# Addressing the human resources crisis: a case study of the Namibian health service

**DOI:** 10.1186/1478-4491-5-1

**Published:** 2007-01-15

**Authors:** Willy McCourt, Magda Awases

**Affiliations:** 1 Institute for Development Policy and Management, University of Manchester, Harold Hankins Building, Precinct Centre, Oxford Road, Manchester M13 9QS UK; 2 World Health Organization, Regional Office for Africa, B.P. 6, Brazzaville, Republic of Congo

## Abstract

**Background:**

This paper addresses an important practical challenge to staff management. In 2000 the United Nations committed themselves to the ambitious targets embodied in the Millennium Development Goals (MDGs). Only five years later, it was clear that poor countries were not on track to achieve them. It was also clear that achieving the three out of the eight MDGs that concern health would only be possible if the appropriate human resources (HR) were in place.

**Methods:**

We use a case study based on semi-structured interview data to explore the steps that Namibia, a country facing severe health problems that include an alarmingly high AIDS infection rate, has taken to manage its health workers.

**Results:**

In the fifteen years since independence, Namibia has patiently built up a relatively good strategic framework for health policy in the context of government policy as a whole, including strong training arrangements at every level of health staffing, and it has brought HIV/AIDS under the strategic umbrella through its National Strategic Plan for HIV/AIDS. Its major weakness is that it has not kept pace with the rise in HIV/AIDS and TB infection: the community counselling service, still at the pilot stage at the time of this study, was the only specific response. That has created a tension between building long-term capacity in a strategic context and responding to the short-term demands of the AIDS and TB crisis, which in turn affects the ability of HR to contribute to improving health outcomes.

**Conclusion:**

It is suggested that countries like Namibia need a new paradigm for staffing their health services. Building on the existing strategic framework, it should target the training of 'mid-level cadres'. Higher-level cadres should take on the role of supporting and monitoring the mid-level cadres. To do that, they will need management training and a performance management framework for staff support and monitoring.

## Background: institutional and HR capacity

Was it realistic for the United Nations to set a target for reducing maternal mortality by three-quarters? For that, along with ambitious targets for child mortality and disease reduction, is what Namibia, the focus of this article, and every other government committed themselves to when they signed the United Nations' Millennium Declaration in September 2000. Reducing child and maternal mortality by 2015, and also the deadly diseases of HIV/AIDS, malaria and TB, is the thrust of three of the eight 'Millennium Development Goals' (MDGs). The Millennium Declaration represents a new direction for development thinking, departing from the 'big private sector and small state' model that was dominant in the 1980s and for most of the 1990s.

Nevertheless, five years on from the Declaration, the UN's own status report showed that little progress on the health Goals had been made in sub-Saharan Africa. Rates of child and maternal mortality, and likewise of malaria and TB infection, all remained stubbornly high – the TB rate was actually increasing. With measles immunization rates staying low and an HIV/AIDS infection rate that was stabilizing but not falling (which in practice meant a growing number of people succumbing to full-blown AIDS), sub-Saharan Africa was worse off than anywhere else in the world [1].

The causes are complex, and beyond the scope of this article. But it is clear that national governments will have to play the leading role in any solution. The 'small state' which has divested or privatized many of its functions is unequal to the task. In fact, the need for governments to take a lead, particularly in the fight against HIV/AIDS, is the very first argument that Francis Fukuyama [2] advances in his recent book *State Building *to persuade his readers, especially in the United States, that the state is fundamentally a good rather than a bad thing, and that we need to strengthen the capacity of weak states rather than cut strong but predatory ones down to size. Treating AIDS, Fukuyama points out, requires a robust public health infrastructure and comprehensive public education. Even if poor countries need help to tackle the problem, they also need institutional capacity of their own, if for no other reason than to channel the resources that might come from outside.

It is also clear, as other contributors to this journal have pointed out, that the way governments manage their health workers is an important part of their institutional capacity. While the budgetary imperative to rein back spending on staff hasn't gone away, we have started to hear a different story in the health sphere from the downsizing narrative that dominated discussion in the 1980s and 1990s, a story about the positive contribution of health workers to public health, rather than their negative drain on public expenditure. For example, we have realized that the more doctors, nurses and midwives there are, the lower maternal, infant and child mortality rates will be [3,4].

Yet despite what we know about the potential contribution of health workers, their actual contribution is woeful. One reason why sub-Saharan Africa is so far from meeting the UN's 2005 Millennium Development Goals for health (which deal with reducing child mortality, improving maternal health and combating HIV/AIDS, malaria and other diseases) is that while Africa is estimated to have 25% of the world's diseases, it has only 1.3% of its health staff [1,5]. In spite of dramatic scaling-up of plans – Malawi, to take one example, was planning at the time of writing for a twenty-fold increase in Anti-Retroviral Therapy (ART), a crucial component of AIDS treatment – the World Health Organization (WHO) was obliged to admit that it would not reach its own '3 by 5' target of giving AIDS drugs to three million people in developing countries by the end of 2005, and staff shortage was again one of the reasons it gave [6]. Botswana's estimate that introducing ART would require 29% more doctors and 8% more nurses than it already had – and no less than 179% more pharmacists – gives an idea of the size of the HR task [7].

It was against this background that Dovlo [8] proposed equipping 'mid-level cadres' to do the work of high-level staff who are a premium, and Wyss [5] proposed the following in his review of the HR implications of the MDGs:

• Recruit more staff from underrepresented groups and regions

• Distribute staff better across rural and urban areas

• Improve performance management

• Improve training

• Improve the policy framework

However, the reality is that while demand for health staff is increasing, the supply is decreasing, and will decrease further if current trends continue. One reason is the 'brain drain' of skilled staff going abroad or moving from the public to the private sector [9]. The OECD estimated in 2002 that no fewer than six out of every hundred health workers in the UK were South Africans [10]. What point was there, for example, in South Africa planning to create 12 000 new health posts when it already had 29 000 that it couldn't fill? Moreover, health worker numbers in Africa have often not kept up with population growth, not least because of the 'downsizing' cutbacks, which made little distinction between health staff and staff in other government activities which are less of a priority and where there might still be scope for reducing numbers.

### Study aims

In the light of the above discussion, this article will analyse the way the Namibian health service manages its staff, focusing on two questions:

• How congruent is staff management with the emerging health care needs that have been outlined above?

• What account is taken of professional 'good practice' in human resource management?

The reason for posing the first question should be self-explanatory in the light of our discussion so far (and, indeed, in the light of the interests of most readers of this journal). The reason for posing the second question is given in the section on 'methods' below.

### The Namibian health service

Namibia has been chosen as a suitable case for exploring the aims of this study because its health service is seen as having done well in providing basic public health facilities and managing its staff, reflected in health indicators that are relatively good in regional terms. In some ways it is not a typical sub-Saharan country. The World Bank classifies it as upper-middle income, largely because of its mineral wealth, and it has a small population of just under two million despite its large area, much of which is desert. Roads and other parts of the infrastructure are very good. But in other ways it is typical. Namibia's human development, at 126^th ^out of 177 countries in 2004, is low, partly because the health of a large section of the population is closer to poorer countries in Africa than national averages suggest. That in turn reflects the apartheid legacy of income inequality and a skewed provision of services. There are also serious gaps in the health infrastructure. Namibia is one of six African countries which have no graduate medical school of their own, so doctors have to be trained abroad or imported [11]. At ministry level there are vestiges of top-down management and bureaucratic habits, as we shall see in the findings presented below.

But the most alarming thing that Namibia has in common with its poor neighbours, and the reason why Namibia's HDI rating is not only low but in relative decline, is the level of HIV/AIDS and TB infection. In 2003 it was estimated that 210 000 people were living with HIV/AIDS, more than one in ten of the population; only Namibia's southern African neighbours – Botswana, Lesotho, South Africa and Swaziland – had higher rates [12]. Knock-on effects included HIV infection of health workers, with prevalence among ante-natal attendants of 8.4 per cent as early as the mid-1990s. The TB infection rate, at 676 for every 100 000 people, was the highest in the world. Moreover, over 45% of TB sufferers were also HIV positive, as TB is the most serious of the opportunistic infections to which HIV carriers are liable to fall victim. As a result, life expectancy, which had been 60 years in 1991, had dropped to 42 by 2002.

Even the relatively sound health infrastructure is creaking under the weight of these problems, as again we shall see in the findings presented below. The precipitous rise in HIV/AIDS and TB has created a dilemma in health strategy and staffing. Unlike some other countries in the region, Namibia has made steady progress in realizing a long-term strategic vision for health care, as we shall see. But its relatively settled structure, an achievement that should not be taken for granted, is now in tension with the need for an urgent response which matches the scale of the HIV and TB crisis. That tension is one of the principal themes of this article.

## Methods

### Research framework

The research framework derives from HR professional 'good practice'. Its principal element is the strategic human resource management (SHRM) model, which is the dominant model among HR specialists, as almost any recent textbook will show. Also, three HR activities have been selected which can be regarded as the building blocks of that model, as well as being prominent in the everyday practice of staff management.

### Strategic human resource management

The SHRM model, which by now is extensively documented on both sides of the Atlantic [13-15], is the main theoretical model on which the study rests. Its principal element is the notion of strategic integration, which refers to aligning staff management systems with organizations' overall strategic objectives (*vertical *integration) and with each other (*horizontal *integration).

Strategic integration implicitly changes the HR specialist's relationship with line managers. The specialist is supposed to design the HR systems that will align with strategic objectives, while the manager is supposed to carry them out. Guest [15] observes that almost all writers say that HR must be managed by line managers: 'HRM,' he says, 'Is too important to be left to the personnel managers.' Thus at the strategic level this study concentrates on the twin questions of 'strategic integration' and 'line manager ownership' of HR. (Line manager ownership is equivalent to what the public management and development literatures call 'administrative deconcentration' or 'managerial devolution'.)

Thus this study operationalizes the SHRM model through two elements: strategic integration and line manager ownership. But we will not confine ourselves to the strategic level. We will also focus on three HR activities: employee selection; performance management and appraisal; and training. We have chosen the first two because:

1. There is good evidence for their effect on organizational performance. The evidence for employee selection is particularly robust and of very long standing [16]. The evidence for performance management, although oblique – it concerns the effect of objective setting and feedback on performance, both central features of performance management [17] – is also robust.

2. There is evidence from previous studies of substantial and interesting national differences in the practice of performance management [18]. Objections from Japan are well known. Companies there prefer to see the team as the unit of production rather than the individual as performance management tends to do [19]. In Europe, Denmark stands out as a country whose democratic workplace relations have held performance management at bay [20].

Our third HR activity, training is also an inherently important element of HR practice. However, there are also specific reasons for focusing on it in the Namibian health service, as we hope will become clear in the discussion below.

### Interview details

The authors gathered data from the headquarters of the Ministry of Health, but also from the central ministries that are 'above' them and from hospitals, clinics and a regional headquarters that are 'below'. Twenty-two semi-structured interviews based on the research framework were held in May and June 2005. The people we interviewed, identified by current and former HR staff in the Ministry of Health and Social Services as carrying a key responsibility for the design of operation of HRM systems, were:

• Two officials in the Department of Public Service Management in the Office of the Prime Minister

• Three officials in the Department of State Accounts in the Ministry of Finance

• The Permanent Secretary of the Ministry of Health and Social Services

• The Under Secretary of the Department of Policy Development and Resource Management, the Department responsible for HR in the Ministry of Health)

• Three current and former officials in the above department

• Staff involved in health training programmes in the University of Namibia

• Staff in the National Health Training Centre, both central and regional

• Managers in a regional health administration

• Managers at Katatura hospital, Windhoek

• Staff in the (USAID-funded) Centers for Disease Control (CDC)

• Staff in two other international agencies active in the health field.

We also ran three focus groups of more junior staff at a central hospital and two clinics, one in the capital and one in a regional headquarters.

## Results

### Strategic human resource management

**Figure 1:**
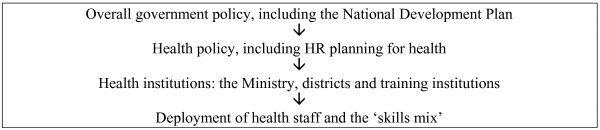


One of the most successful features of the Namibian health service is that it is a relatively well integrated system, not only within the Ministry of Health but across government as a whole, as Figure 1 shows.

The foundation document is Namibia's 'Vision 2030' [16]. It is not unique to Namibia: it derives ultimately from Malaysia's well-known 'Vision 2020', and both Botswana and Lesotho in the region have versions of their own. One level below it is the National Development Plan process; Namibia is now in the period of its second Plan (2001–2006]. One level below that again is health policy. The basic framework was laid down in 1990 at the time of independence in *Towards achieving health for all Namibians: A policy statement*, which the government reviewed in 1997. The thrust of health policy is reflected in the five-year *Human resource strategic plan 2000/1–2004/5*. There is also a *'Public Service Charter' *for government as a whole, modelled on the *'Charter for the Public Service in Africa' *(in which Namibia played a leading role) and ultimately on the UK's *'Citizen's Charters'*. It sets out standards of behaviour, including courtesy to clients, to which public servants should cleave. There is also written evidence of 'strategic integration', in the sense of a fit between overall strategy and the way that staff are managed: HR issues do get addressed in the context of strategic programmes. There are, for example, sections on HR in both the *National strategic plan on HIV/AIDS 2004–2009 *and the *Guidelines for the prevention of mother-to-child transmission of HIV (PMTC)*.

Such documents have their limitations. Experts on strategic management and project planning like Mintzberg [17] and Korten [18] have long emphasized that it is the quality of strategic thinking, rather than the content of strategic documents with their inevitably short shelf-life, that is crucial: 'blueprint project' designs are usually more impressive on paper than in practice. The full 30-year HR Plan, for example, was never finalized because of a ministry restructuring which overtook it. Moreover, strategy implementation is affected by the apartheid legacy which has not wholly been shaken off. A senior official talked about 'Inheritance from the apartheid days ... After independence the old bureaucrats indoctrinated the new people.' Implementation is also affected by the pressure under which many staff are working. Focus groups which we conducted with hospital and clinic staff in both the capital and a district headquarters showed that as well as heroic dedication, staff are often exhausted and demoralized:

'You get so demotivated because you're doing the same thing all the time, but have no time to ask patients if they are really OK. In the old days – five years ago – TB patients were healthy, just needing medication once a day. Now they need to be bathed, turned ...'

But having made those qualifications, there are positive features to be noted. What is arguably more important than any particular policy document is the sense one gets of a health service that knows where it is going, and has been prepared to revise and restructure when necessary to get there. One senior expatriate commented that 'The environment here is decisive ... an excellent conduit through which to roll out programmes.' Another believed that Namibia's good record 'has to do with governance and leadership at the higher level ... There is a vision and it's really used.' The Ministry of Health has restructured several times, most dramatically just after independence, but also with the revision of the staff establishment in 1996, and later through a major decentralization to four regional directorates and thirteen regions, each time in an attempt to align the health service more closely with government health objectives.

However, Namibia's relatively strong strategic framework still had one fundamental deficiency. The *National Strategic Plan on HIV/AIDS *contained no estimate of the current level of HIV infection, despite being two hundred pages long and covering every relevant area of government activity, and, crucially, it stopped short of setting a target for reducing infection rates, or of showing how its hundreds of proposed activities would reduce the number of people who were infected or who would die. In the public management jargon, those activities were 'outputs' rather than 'outcomes'; means rather than ends [19]. (We hope it is not pedantic to point out that the Plan incorrectly uses the word 'outcome' to refer to activities such as increasing condom use rather than the results that the activities are designed to obtain.) When only eight months later it did set the target which the standard format for applications to the Global Fund to Fight AIDS, Tuberculosis and Malaria required [20], it was the modest one of reducing HIV prevalence among 'younger age groups' by 5% over the five years of the project – which at best would reduce overall prevalence from 10% to 9.5% of the population.

The government's reluctance to offer up an ambitious target as a hostage to fortune is understandable, especially when it needed to keep a lid on public spending at the same time (and we will see that the Ministry of Health could not persuade the government to give it the money to train the number of enrolled nurses that it believed it needed). After all, even the much admired New Zealand government of the late 1990s fought shy of setting outcome targets. The new Labour government in the UK in its post-election euphoria did set itself no fewer than 600 such targets, which the responsible minister was prescient to describe as '600 rods for our own back' at the press launch in 1999 [19].

But the government's reluctance meant that its actions would almost certainly not rise to the scale of the problem. It also meant that the donors, and particularly American donors in whose aid strategies the HIV/AIDS campaign is a central plank, were in the strange position of displaying a greater sense of urgency than the government, and of trying to persuade the government to speed up its response, even if that entailed cutting across the well established health strategy. Thus arose the tension between long-and short-term priorities, including in the area of HR, to which we have already alluded, and to which we will return.

### HR at government and ministry level

The requirement to advertise almost all vacancies externally which comes from the government's commitment to affirmative action (see below), and the deliberate delay in filling vacancies which results from the overall budget deficit (7.5% of GDP in 2003/4) are just two examples of the impact of central government on health staffing. For as long as governments manage their public servants centrally, for better or worse, tedious restrictions like these will persist.

At ministry level there was an anachronistic split which no one in the ministry defended between its departments of Human Resource Management and Human Resource Development, with HRM mostly playing the mundane administrative role of handling leave forms and other HR housekeeping chores, and HRD dealing with training, but also taking occasional initiatives such as marketing careers in health to school and college leavers. To some extent strategic tasks fell between the two stools, so that a strategic HR response to health policy objectives tended to come from the overall health policymakers rather than from the HR function. One informant with experience of both sides said that 'You will find a HR response to HIV/AIDS in the HIV/AIDS division, but not in the HR division. It's usually driven by the programmes.'

### Line manager ownership

Despite central controls, staff management is still relatively decentralized in Namibia. Ministries appoint their own staff up to and including the permanent secretaries who are the official heads of ministries, and it is staff at local level who make the appointments, with HR staff merely acting as secretaries. Politicians seem to keep out of staff appointments except, of course, the very senior ones that only they can make. Even then, their influence is marked only in 'sensitive' ministries like Defence. Politicians seem to disagree with their Sri Lankan counterparts that the only way to get senior officials to implement your policies is to appoint them yourself. On the other hand, there is no sense either of Sri Lanka's ideological divide between politicians and officials [21,26].

The central controls that persist were resented most fiercely at the sharp end. If a matron resigns in a district, a senior district officer complained, the request to replace her has to go first to headquarters, then to Finance and OPM (despite OPM's claim that these appointments have been decentralized). Up to three months are lost before the post can even be advertised. At the lowest level, a local health centre manager was beyond resentment: 'We just take what we're given.'

However, at the other end of the system, Finance officials were surprisingly relaxed about giving more power to ministries: 'I'm neutral on corruption as between a centralised and a decentralized system,' said one of them, who believed that the only way that ministries would ever learn to manage budgets was by being made to. With a move to 'programme budgeting' trailed in the finance minister's 2005 budget speech, it looked likely that ministries would soon have greater freedom – with the notable exception of pay: previous experience with performance-related pay suggested that this would create a temptation that some managers would not be able to resist: 'It wouldn't be a good thing if accounting officers could authorize (pay), it would lead to corruption,' according to a central HR official.

### Employee selection

The driving force behind Namibia's employee selection procedures is affirmative action. As in South Africa, Namibia's newly independent government inherited a society with a (white) settler community that was richer and better educated than the indigenous majority. However, the South West African People's Organization (SWAPO) in Namibia espoused non-racialism and had a small but significant white membership, just like South Africa's African National Congress (ANC). It wanted to 'redress the racial balance', but it also wanted to abolish the race discrimination that was apartheid's hateful essence.

Clearly these two desires were contradictory. The Ministry of Health's Personnel Procedures Manual defined affirmative action as

'a process whereby action is taken to end unfair discrimination ... without evoking perceptions of reverse discrimination among certain groups.'

But the Manual also stated that

'Government expects managers to ... consider primarily disadvantaged persons when they recruit new staff members' [27].

It is not clear how 'advantaged persons' could be ruled out without evoking the perception of reverse discrimination. To be sure, the government could only change the composition of the workforce over any politically realistic timescale by preferring relatively unqualified black candidates over white candidates, in a context where whites historically have been better educated than blacks. On the other hand, doing so drove a coach and horses through the ideology of non-discrimination. Hence the contradiction.

However, Namibian practice was still relatively transparent. Remarkably and almost uniquely among poor and rich countries alike, the affirmative action imperative meant that posts at every level were open to competition from outside, leading managers to complain that they could not use discretionary promotion as a way of retaining staff. Affirmative action has also led to explicit procedures, again as in South Africa. There was written guidance for managers on how to draw up job descriptions, and managers were expected to keep records of their reasons for shortlisting and appointing candidates. Rejected candidates could appeal, and the appeals procedure had sharp teeth: appellants were successful in no fewer than 20 of the 25 appeals brought in 2003/4 across government as a whole [28].

Strictly speaking, what organizational psychologists call a 'validation study' would be needed to demonstrate that these elaborate procedures have made a difference to the quality of staff appointed and the work they do. But experience elsewhere gives us good reasons to believe that they will. Apart from that, transparent procedures like these will tend to increase citizens' confidence in the way government manages its staff on their behalf [28].

### Performance management

Following independence a performance management scheme which included a performance management pay element was introduced across government as a whole. Assessment by managers was intensive, taking place once a quarter. It was possible for good performers to get two salary increments ('notches') or even three in a single year. However, the scheme foundered in 1998 in a welter of accusations of nepotism: *'The old scheme failed because it was too subjective,' *said one informant; *'friends of bosses got notches' *said another. There were also suggestions of what assessment experts call 'leniency bias' [30]: *'Managers were giving everyone the notch' *because they were *'just afraid to say no'*. The scheme as a whole was seen as '*arbitrary'*, leading to complaints from the powerful trade unions. At the time of our interviews a new 'Performance Management System' was being piloted in the Ministries of Labour and Agriculture after a lapse of seven years. Significantly, the emphasis in the new scheme was on competencies; managers would have no power over pay.

Overall, Namibia's performance management experiments have followed a similar trajectory to those in other developing countries such as Mauritius and Malaysia [31,32]: the belated introduction of a scheme with a pay element, intended to introduce the culture of performance, quickly falling foul of particularistic tendencies in the broader society, but also of the sheer inability of managers to deal fairly with staff; and the tentative re-introduction after a cooling-off period of a watered-down scheme which emphasized employee development rather than pay, but which still had to prove its worth.

### Training and the health 'skills mix'

The context for our discussion of training is the mixed fortunes of training in development thinking since the 1960s. In the period between independence and 1990, improving public staff management, 'capacity building' [28] and even developing public administration became all but synonymous with training public employees once the basic state structures were in place. There were two principal vehicles for this. The first was the national administrative staff colleges like INTAN in Malaysia and MIPAM in Mauritius which newly independent countries set up to provide pre- and in-service training for their civil servants. The second was the overseas scholarship programmes which took up a hefty share of aid agency and government training budgets. All this activity was based on a simple 'skills deficit' premise: developing country governments were seen as underperforming at least in part because their staff lacked skills, and training was seen as *the *way of making up the deficit.

But training has fallen from grace. The training-led model of public staff management began to decay in the late 1980s, for four reasons. The first was the pressure to reduce public spending which resulted from the two oil price shocks of the 1970s; training is notoriously one of the first spending items which organizations everywhere cut when they get in trouble. The second reason followed from the first: the absurdity of training new staff at the same time as trying to save money by 'downsizing' the staff that governments already had. The third, insidious reason was the loss of belief in training as a form of development assistance. It had become equated with 'trainingism': training as an end in itself, serving the vested interest of training providers and of the trainees who pocketed what they saved from their training allowances [34]. When the critique was applied to official UK aid spending on training, it was a convenient pretext for a Conservative government that was happy to see aid spending decline to withdraw almost entirely from stand-alone training [35]. Finally, spending less on training was the opportunity cost of the recognition that training was not the be-all and end-all of public management: to some extent spending migrated to support for programme budgeting, 'results oriented management', performance management and so on. Those who suggested that training could still be effective if it was better targeted and more 'strategic' such as McCourt and Sola [36] were swimming against a powerful tide.

The health service in Namibia has bucked this trend. To understand why, we need first to grasp the concept of what Buchan and Dal Poz have called the 'skills mix': the appropriate mixture of skills and jobs that health services need. For example, they suggest that 'there is unrealized scope ... for extending the use of nursing staff and for further development of care delivery led by nurses/midwives' at the expense of tasks currently done by doctors [37,8].

**Figure 2:**
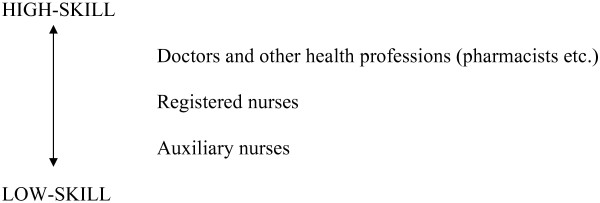


Figure 2 shows what Namibia's health staffing hierarchy looked like at independence.

This structure created two problems. The first was the skill deficiencies caused by the gaps in the structure. At the 'top', there was no provision for clinical specialization, and at the 'bottom', auxiliary nurses were mostly untrained and could not provide specialized care. Throughout the structure there were staff shortages, caused by the delay in providing high-skills training, and also by loss of high-skill staff who were attractive to the private sector, and likewise by movement from rural to urban areas.

**Figure 3:**
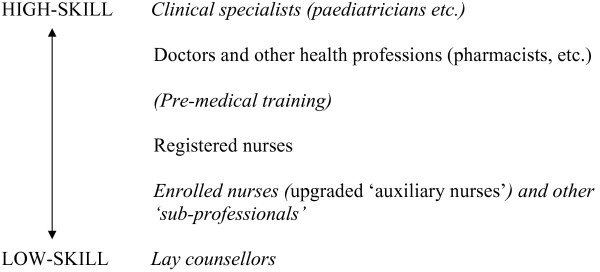


Figure 3 shows what state the staffing structure was in at the time of this study, after 15 years of independence.

This structure was the outcome of a large number of separate initiatives. The most impressive was probably the scheme to upgrade auxiliary nurses to enrolled nurses. Described as a 'milestone' in Namibian health care, it had its origin in the move to primary health care following independence, and a 1992 report called *Integrated health care delivery: the challenge of implementation*, prepared with support from WHO. The next step was to recognize that a system of 'sub-professional' training was the most relevant and affordable way of supporting national primary health care priorities. Donor support was important, but once again it supported indigenous plans rather than substituting for them. An example of this is that the enrolled nurse training structure was inaugurated with skeleton staffing before donor resources began to flow. Consequently, donor resources were effective through simply adding to a structure that already existed, for example by providing funds for supplementary tutors to be appointed.

A National Health Training Centre was established in Windhoek, with four regional centres around the country, a network described by a donor-funded expatriate as a 'phenomenal resource'. Its courses had been running long enough at the time of writing for positive results to be evident. There was a very high training completion rate: 97% in one regional centre. In at least one northern district, patient mortality rates improved following enrolled nurse deployment. Staff themselves were entirely positive about the scheme, especially the former auxiliary nurses who were its main beneficiaries: 'It was an eye-opener – we thought we were doing the correct things.' 'Now we can manage the clinic alone, there is more responsibility. Salary-wise, it is better too.' One worker put the advantages in a homely way: 'You dusted off for the sake of dusting off, now we know why.' Finally, it was perhaps as positive a sign as any that private clinics and hospitals were willing to employ some of the enrolled nurses themselves. With the fears of better qualified colleagues that the nurses' short training would not give them the necessary skills having proved groundless, the enrolled nurses were becoming an accepted component of the 'skills mix'. Despite problems such as nurses getting out of their depth, especially when their managers failed to support them properly, everyone agreed that this initiative had been a success.

Action to improve staffing in the medium term had been taken at other levels of the staffing hierarchy as well. There was a programme of pre-medical training to make up for school-leavers' poor performance in science subjects, and postgraduate training in clinical specializations such as obstetrics. Equally important was the six-week programme of training for a new cadre of 'community counsellors' who would have time to explain to patients the ramifications of treatment in a way that doctors and nurses couldn't do. Following deployment of trained counsellors, enrolment in Mother to Child Transmission programmes, designed to prevent mothers from passing on AIDS to their babies, jumped from 8.5% to 85% of patients.

Sidestepping the staffing establishment was an element in the success of both the enrolled nurse and the counsellor training programmes. The point of a staffing establishment is to keep a lid on spending by making it hard for ministries to create posts, but it makes it hard to respond quickly to a new need. This was avoided for the enrolled nurses by giving trainees the status of students rather than employees so that they would not be part of the establishment. Similarly, the counselling scheme was managed by the Red Cross, and counsellors were formally Red Cross (not government) employees.

Altering the 'skills mix' by lowering the fences between the traditional health professions is an example of what has been called 'functional flexibility' in the context of the new SHRM model. It is supported by evidence that using nurses and care assistants to do what has traditionally been doctors' and nurses' work is not at the expense of quality of care [37].

These changes aligned staffing much more closely with the health priorities of the new government. This, we suggest, is an example of the Mintzbergian 'strategic thinking' model of strategy rather than the blueprint model (see also Peters and Waterman [38]). What was crucial was the shared understanding about the reorientation of the health system that would be needed to move away from apartheid provision and towards provision for all Namibia's citizens, and from curative-based to preventative medicine, with all that this reorientation entailed for HR. This again was a result of the focusing of minds that came with the liberation struggle. Without it, the health service would have been like an anthill when the Queen has been removed, and no strategy document, however elaborate, would have been a substitute.

## Discussion: HR and the health crisis

### Characterizing HRM in the Namibian health service

The study results provide a picture of a health service where at the strategic level there is a relatively strong strategic framework, but where key strategic challenges, notably the challenge of HIV/AIDS, have been fudged, since strategic aspirations have only partially been translated into operational objectives. This is a major weakness. The premise on which this article is based – and equally on which the journal in which it appears was founded – is that the human resource is a major but neglected means of improving the health of the citizens of poor countries. That in turn requires HR to be aligned with the health challenges that countries face. On any reckoning, the fight against HIV/AIDS, and also TB, are major challenges, yet staffing strategy in Namibia has only partly addressed them.

At the operational level the picture is mixed. The government's steady post-independence determination to move from a curative to a preventative orientation is reflected in the 'phenomenal' network of training institutes, and in some imaginative and flexible training provision which managed to circumvent the government's monolithic staffing structure. Similarly, the government's consistent commitment to affirmative action means that employee selection is relatively sophisticated, not only in professional HR terms but in terms of 'voice', through a strong appeals procedure which provides vital feedback on the quality of selection decisions (albeit feedback that individual managers might prefer not to have). Conversely, efforts to manage staff performance have ground to a halt, stymied by particularistic tendencies in the wider society and by the partly related opposition of the powerful trade unions. However, these mixed results do have one overarching factor in common: they are all alike grounded in indigenous factors of political economy. In the case of the government's commitment to preventative care and affirmative action, those factors have a positive effect on HRM; in the case of the particularistic tendencies and the strength of the trade unions, the effect is negative.

### Strategy, urgency and commitment

At the start of this article we noted how the Millennium Development Goals represent a new departure in development thinking, and how public staff management has come to be seen as central to realizing them, especially in health. This article has explored what that means in practice for one sub-Saharan country in Africa. Namibia has patiently built up a strategic framework for health policy in the context of government policy as a whole, including strong training arrangements at every level of health staffing, and it has brought HIV/AIDS under the strategic umbrella through its National Strategic Plan for HIV/AIDS. The framework is imperfect, but expatriates who know other African countries agreed that it works relatively well. Its major weakness is that it has not kept pace with the rise in HIV/AIDS and TB infection: the community counselling service, still at the pilot stage at the time of this study, was the only specific response. That is the source of the tension between building long-term capacity in a strategic context and responding to the short-term demands of the AIDS and TB crisis.

In a way the tension ought to be a false one, for the crisis has not come out of a clear blue sky: it was as long ago as 1986 that Namibia's first four cases of AIDS were identified. Strategic management is by definition a long-term activity, but the period over which AIDS and the associated rise in TB infection have developed comfortably crosses the strategic time threshold. AIDS and TB have become an urgent issue because it is only very recently that governments in southern Africa, including Namibia's, have begun to follow Uganda's example in being candid with their citizens and then acting decisively (Uganda was the ultimate model for Namibia's counselling programme). As a result, infection rates have been allowed to creep upwards. In Uganda, the percentage of pregnant women attending antenatal clinics who were HIV positive went down from 31% to 14% between 1992 and 1998. In Namibia, it went up from 4.2% to 17.4% over the same period, and by 2002 it had risen further to 22% – and this in a country that is much richer than Uganda, showing that lack of money is only part of the story.

The dilemma that Namibia was facing at the time of writing, in its overall health strategy as well as in the HR approach which is a particular manifestation of that strategy, was to find an adequate response to the particular problems of HIV/AIDS and TB without damaging the strategic framework that it had taken so much trouble to set up. On the one hand, policies have succeeded in Namibia as much because of the framework in which they are set as because of the policies themselves. Namibia's health strategy experience shows the futility of 'cherry-picking' individual practices out of the framework in which they have arisen, even when they are targeted at very urgent health problems like AIDS. On the other hand, the very solidity of that framework has militated against responding to a pandemic which has no respect for strategic timetables. The picture is complicated by the government's emphasis on the framework being in conflict with donors' emphasis on the problems.

Fundamentally, we suggest, this is a problem of political commitment. It is significant that WHO [39] believes that the crucial factor in Uganda's success was political commitment. The global report of UNAIDS [12] calls for 'strong leadership and concrete commitment from all sectors of government and society', and it may be equally significant that a whole section is devoted to the same topic in Namibia's AIDS Strategy document. If we apply the model of political commitment developed by McCourt [40], we can see that the 'antecedents' of commitment in the form of political and administrative capacity are present, but that the 'elements' are defective in one important way. While Namibia's commitment to tackling the pandemics is voluntary, explicit and public, it is not sufficiently challenging in that, as we saw, no target has been set for reducing the scale of infection.

It is outside the scope of this article to address the politics that must underpin any public staffing initiative (see Alarkoubi and McCourt [41]). However, the World Bank [42] has noted in its *Public Expenditure Management Handbook *that 'In many respects, political will is a function of the quality of advice provided to politicians'. Officials need to show politicians that there is a target for reducing infection rates which it would be realistic to set. That in turn will mean showing that the resources are in place to meet the target. This applies across the strategic board, as it were, but it applies acutely in HR, for while attention has focused on access to drugs like Anti-Retrovirals, the human resource will also be needed to administer those drugs.

### Relationship with donors

The relationship with donors, touched on in the last section, is a necessary factor in our discussion. As a relatively wealthy country, Namibia does not rely heavily on donors, who in 2003 provided only 3.4% of GDP (compared to 13.1% in Tanzania). Partly for this reason, Namibia has been successful in ensuring that donors support indigenous priorities: 'When you're not aid-dependent, you can stay in charge.' High-level meetings with donors were chaired by the minister, and donor proposals were worked on intensively within the ministry at the planning stage, so that pressure to import pet schemes which donors have implemented in other countries was resisted. Despite this, the Namibian side still had to assert itself from time to time. This was especially true, it appeared, in dealing with American donors, who 'can be quite hard to keep under control', having domestically generated objectives of their own which cut across the Ministry's – including, one senior official claimed, a preference for expensive drugs manufactured by American companies rather than generic substitutes. (It may be relevant that during the writing of this article the US Ambassador to the United Nations was pressing to have the following phrase deleted from the draft of a United Nations summit agreement: 'encourage pharmaceutical companies to make anti-retroviral drugs affordable and accessible in Africa' [43].)

From an outside perspective, Namibia sometimes seemed too ready to resent donors suggesting models from other countries. But it is in keeping with an emphasis on indigenous ownership of staff management initiatives, and there was concrete evidence of its value. The Ministry of Health has used its position of strength to face down donors' preference for freestanding projects and to harness their support to recruiting more staff and training more health workers than they would otherwise have been able to do. One example was the 'parallel hiring scheme' run by the American CDC with support from USAID, where additional staff were appointed on the standard ministry terms. At the time of writing 13 doctors, 10 nurses, 10 pharmacists and 10 data clerks had been recruited, and there was approval to go up to 27, 15, 15 and 15 respectively. Similarly, funding from CDC made it possible for the number of nurses trained to increase dramatically from the 50 that the Ministry of Education was prepared to fund to around the 200 that the Ministry believed it needed to train: 'CDC bridged the gap.'

This style of support incurred a minimum transaction cost in money, time and expertise, because the government's administrative structures already existed. It should not be a surprise that Ministry staff seemed to appreciate it more than any other kind of donor assistance.

## Conclusion: towards a new HR paradigm

In this concluding section we discuss, finally, what HR action should be a priority, both in Namibia and in other countries. The fundamental strategic need is to estimate the staffing that will be needed to scale up AIDS and TB provision and move towards the MDGs. This is a need that Namibia has only partly addressed. Assuming more ambitious targets than the current ones for reducing infection rates, there should first be an estimate of how far the current staffing paradigm can be extended; in other words, how much scope there is to fill more vacancies through additional funding from central government and donors, as has already been done.

However, it seems clear that even with the extra funds that were scheduled to come from the Global Fund, debt relief and other initiatives, countries like Namibia will need a radically different staffing paradigm if they are to go beyond sheer wishful thinking or the fudged response that Namibia has made up to now. We saw that Botswana estimated that it would need, among other things, 179% more pharmacists to administer ART. But this was a mechanical and completely unrealistic extrapolation from current arrangements: there are just not enough pharmacists to go round.

What shape might a new paradigm take? It would have to come from within the health system, where both the necessary knowledge and the basic ownership of the problem reside. However, we may be able to perceive the shape of a solution from what we know already. First, it must be accommodated within the basic strategic framework which has been built up over 15 years and should not be destabilized. Second, it should target middle and lower levels in the staffing hierarchy. We know that African countries are finding it hard to recruit and retain the highly qualified staff whose training is so time-consuming and expensive. We also know that the experience of using what Dovlo has called 'mid-level cadres' to substitute for those staff is positive. Therefore it seems appropriate to use job analysis and competence development to identify the precise skills that the new treatments will require, and to provide them to trainees at the lowest possible level, equivalent to Namibia's enrolled nurses and community counsellors. There is in every African country a large pool of unemployed people, notably recent school leavers, who have only basic qualifications. They can be trained quickly and cheaply, their training will not be sophisticated enough for other countries to want to poach them, and they are more likely to have roots in rural and poor areas that will make them want to stay there. There are probably also existing staff like Namibia's auxiliary nurses whose skills can be upgraded. There may also be public employees in low priority areas who can be retrained and redeployed.

Namibia's experience shows that such trainees need professional support and supervision. Staff at higher levels will need to take extra supervisory duties. 137 health staff had successfully completed Certificates in Management up to the time of writing in Namibia. However, the more urgent need is for short, intensive development programmes that will provide management skills that can be used immediately. It is very desirable that there is a performance management framework within which those skills can be used.

This study concludes with the possibility of a new paradigm for HR in governments like Namibia's which need to respond to the health crisis, and to move towards the MDGs to which most governments have committed themselves. It is absolutely in keeping with what we know about strategic management that a new strategic direction will mean a new approach to HR (and also to public management in general). It will include the elements that this study has focused on: a strategic framework, since governments should proceed from a strategic sense of overall HR needs in the light of national policy objectives; line manager ownership, since lower level and relatively unqualified staff will have greater supervision needs; good employee selection to identify candidates who may lack normal qualification badges of achievement but who have some relevant skills and the potential to acquire more; and a system of performance management for managers to supervise and support these new staff. But it is likely to give pride of place to the deployment and pre-service training of middle- and lower-level health workers: training, which has been out of fashion for 15 years in development management, regains the importance it had in the early independence period. There is also room for creativity in organizational arrangements: Namibia's delegation of its counselling scheme to an NGO, the Red Cross, may be a straw in the wind.

## Competing interests

The author(s) declare that they have no competing interests.
